# Ring-Shaped Microlanes and Chemical Barriers as a Platform for Probing Single-Cell Migration

**DOI:** 10.1038/srep26858

**Published:** 2016-05-31

**Authors:** Christoph Schreiber, Felix J. Segerer, Ernst Wagner, Andreas Roidl, Joachim O. Rädler

**Affiliations:** 1 Faculty of Physics and Center for NanoScience, Ludwig-Maximilians-Universität München, Geschwister-Scholl-Platz 1, D-80539 Munich, Germany; 2 Department of Pharmacy, Center for System-based Drug Research, Ludwig-Maximilians-Universität München, Butenandtstraße 5-13, Building D, 81377 Munich, Germany

## Abstract

Quantification and discrimination of pharmaceutical and disease-related effects on cell migration requires detailed characterization of single-cell motility. In this context, micropatterned substrates that constrain cells within defined geometries facilitate quantitative readout of locomotion. Here, we study quasi-one-dimensional cell migration in ring-shaped microlanes. We observe bimodal behavior in form of alternating states of directional migration (run state) and reorientation (rest state). Both states show exponential lifetime distributions with characteristic persistence times, which, together with the cell velocity in the run state, provide a set of parameters that succinctly describe cell motion. By introducing PEGylated barriers of different widths into the lane, we extend this description by quantifying the effects of abrupt changes in substrate chemistry on migrating cells. The transit probability decreases exponentially as a function of barrier width, thus specifying a characteristic penetration depth of the leading lamellipodia. Applying this fingerprint-like characterization of cell motion, we compare different cell lines, and demonstrate that the cancer drug candidate salinomycin affects transit probability and resting time, but not run time or run velocity. Hence, the presented assay allows to assess multiple migration-related parameters, permits detailed characterization of cell motility, and has potential applications in cell biology and advanced drug screening.

Migrating cells play a pivotal role in morphogenesis^1^, immune responses^2^, and cancer metastasis^3^. Their style of motion, often assigned as crawling, is powered by complex cytoskeletal rearrangements that deform and propel the cell. On solid surfaces, eukaryotic cells extend protrusions, which attach to the substrate and are then actively retracted, thus dragging the cell forward. The formation of the leading protrusion of a migrating cell, the lamellipodium, is driven by actin polymerization, while adhesion and contraction are predominantly regulated by integrin-based focal adhesions and the actomyosin apparatus^4^,^5^. Coupling of focal adhesion complexes to the cytoskeletal network in turn reinforces actin assembly and hence lamellipodia extension^6^. The complex interplay between actomyosin contractility and focal adhesions, which are capable of sensing and transducing chemical and mechanical cues in the extracellular environment, renders the cell sensitive to external stimuli such as the composition and rigidity of the extracellular matrix (ECM) and the underlying substrate^7^,^8^.

In recent studies, various theoretical models for cell migration have been proposed and implemented. These implementations range from molecular level approaches, which describe cell migration in terms of internal reaction diffusion dynamics^9^,^10^,^11^ to coarse grained approaches in which individual cells are resembled by sets of pixels^12^,^13^,^14^ or interacting, self-propelled geometrical objects^15^,^16^,^17^. Many of these models are able to reproduce the basic features of cell migration. However, in order to advance our understanding, the migratory patterns emerging *in silico* need to be compared to those observed *in vitro*. To this end, the migratory behavior of a cell, which is generally dependent on cell type as well as environment, needs to be assessed within a quantitative framework.

Moreover, deviations from the typical migratory behavior of a cell can often be an indicator for pathological conditions or pharmaceutical influences. One of the most prominent examples of a drastic change in migratory phenotype is the epithelial-to-mesenchymal transition (EMT). In this process, epithelial cells loose the contacts with their neighbors and acquire invasive properties, such as increased motility and the ability to overcome barriers^18^. Since EMT is assumed to play a crucial role in cancer metastasis^19^, drugs used in cancer therapy often aim to downregulate cell motility^20^. One such example is the anti-cancer drug candidate salinomycin^21^, which exerts an anti-migratory effect by modulating calcium dynamics in cells^22^. Hence, in order to detect anomalies in migration behavior, suitable metrics must be found that characterize cell motility and its relationship to environmental conditions. The development of such metrics is consequently of fundamental importance in pathology and drug screening^23^,^24^,^25^. However, extracting characteristic and meaningful parameters from time-lapse movies of individual cells is a challenging task.

The approach most frequently used to analyze single-cell motility involves tracking cells on a two-dimensional (2D) surface, and calculating their mean velocity or employing the persistent-random-walk (PRW) model to extract the directional persistence^26^,^27^. In recent studies, however, it was observed that the migratory path described by various cell lines does not follow the standard PRW model^28^,^29^,^30^. Furthermore, global measures like the mean velocity yield only limited insight into the heterogeneous migration dynamics of living cells. Phenomenological models that include dynamic switching between two different modes of migration often capture the observed migration patterns better, and hence provide more detailed insights into cellular behavior^31^,^32^,^33^. The emergence of such bimodality is predicted by theoretical approaches which assume a coupling between actin dynamics and the diffusion of cell internal polarity cues^34^.

In order to simplify the assessment and comparison of motility-related parameters, micropatterning techniques can be exploited to create standardized cell environments and restrict cell migration to defined geometries^35^,^36^,^37^. The resulting cellular shapes and dynamics can in turn be compared to theoretical modeling^38^. In fact, micropatterned environments have proven to be well suited for the study of migration, both at the single-cell level^39^,^40^ and on the scale of small and larger cell assemblies^14^,^41^,^42^. In particular, racetrack patterns that restrict cell migration to one dimension (1D) have proven useful for studying migration-related properties in a standardized and automated manner, and provided motility parameters for many cell types^34^,^43^,^44^,^45^. The standardized framework of micropatterns additionally enables the systematic study of the significant heterogeneity in the migratory behavior of single cells of the same type^44^. Another advantage of micropatterning, which has been highlighted to a lesser extent, is that spatially well-defined junctions between different substrates can be created. In general, the nature of the substrate is a crucial factor in cell adhesion and migration and is known to affect migration of different cell types^46^,^47^,^48^. The defined interfaces created by micropatterning can be exploited to systematically study the impact of an abrupt change in substrate composition on a migrating cell. If the migration machinery of a cell is suddenly perturbed, the resulting response can yield significant insights into its intrinsic properties. However, how exactly migrating cells respond to transitions in substrate composition is unclear, and a combined migration assay that allows for quantification of migration and cell interactions with chemical interfaces in micropatterns has yet to be established.

In this work, we characterize cell migration in ring-shaped microlanes and address the question of how migration is affected when cells are guided head-on into barriers consisting of chemically modified areas. In a first set of experiments, we analyze 1D migration in the lane and find a bimodal behavior comprising distinct phases of directed migration (run states) interrupted by phases of reorientation (rest states). By employing a change-point analysis, which detects switching between both states, we find the persistence times of both to be exponentially distributed and evaluate the characteristic persistence times. In a further set of experiments, we insert a PEGylated barrier into the lane and systematically investigate the cells’ interaction with the chemical interface. These encounters have two possible outcomes: Cells either go into reverse at the barrier or they traverse it. The probability that a cell crosses a barrier decreases exponentially with barrier width. Our microarray-based approach thus yields a five-parameter description of migration akin to a migratory fingerprint. We utilize this assay to quantify the migratory behavior of the mesenchymal MDA-MB-436 relative to the more epithelial HuH-7 cancer cell line, and determine the effects of the anti-migratory cancer drug candidate salinomycin.

## Results and Discussion

### Single-cell migration in ring-shaped microlanes

**Figure 1:**
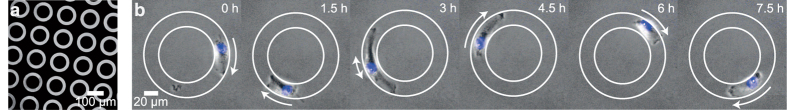
Cell migration in ring-shaped micropatterns. (**a**) Fluorescence image of the pattern consisting of labeled fibronectin (bright) and PLL-PEG (dark). (**b**) Time-lapse series of phase-contrast images of a MDA-MB-436 cell migrating along a ring-shaped lane. The fluorescently labeled nucleus is indicated in blue. Cell morphologies predominantly fall into two classes: asymmetrically polarized cells with a single lamellipodium at the leading edge and the nucleus located at the posterior end, and elongated cells with two, symmetrically positioned lamellipodia.

In order to study single-cell migration in a standardized and well defined environment, we generated arrays of quasi-one-dimensional micropatterns in the form of ring-shaped, fibronectin-coated microlanes (Fig. 1a). For the appropriate pattern radius, a tradeoff had to be made. On the one hand, small radii enable a high pattern density and hence increase the statistical output. On the other hand, larger radii provide a lower curvature of the lane hence avoiding artificially curved cell shapes and hinder cells to span the passivated center of the ring. We consequently chose a radius of 50 μm for all experiments. The width of the lane was chosen to be 20 μm. We observed that cells confined in a lane of this width exhibited a morphology closely resembling that of cells migrating on open 2D surfaces, but were sufficiently constrained to ensure that migration was directed along the lane only (Fig. 1b). On such patterns, cells migrate in circles along the lane, showing quasi-one-dimensional motion. In contrast to straight stripes, the circular shape has the advantage that cells that move over a distance of several millimeters still remain in the same field of view and cannot collide with neighboring cells. Consequently, on the order of 100 separately migrating cells can be analyzed in parallel over time periods of several days. In addition, this avoids a bias in estimates of the mean velocities resulting from an earlier loss of faster cells from the field of view compared to slower cells.

**Figure 2:**
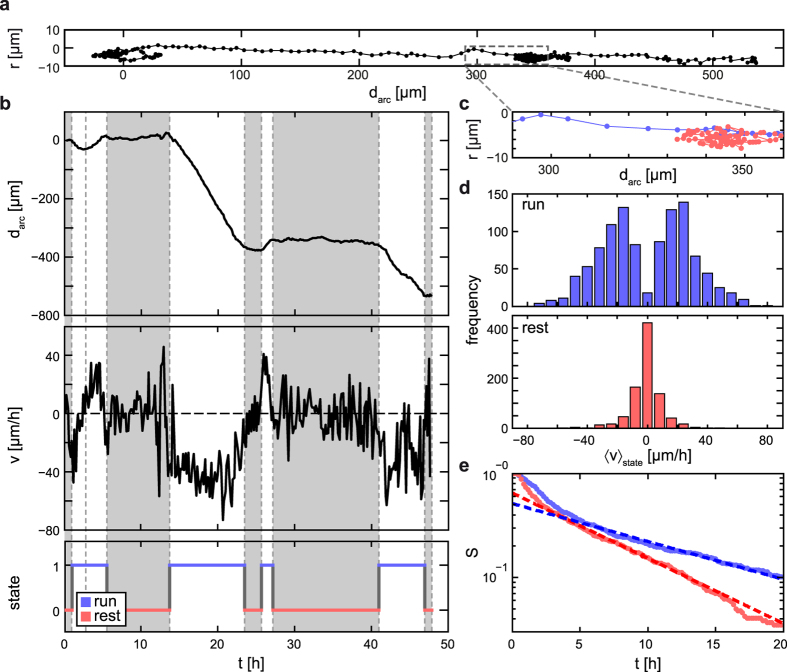
Bimodal analysis of cell tracks. (**a**) Typical trajectory of an MDA-MB-436 cell confined within a ring-shaped pattern. The radial cell position, *r*, is plotted against the tangential position, *d*_*tang*_ = *R* · *φ*, calculated as the product of the mean lane radius, *R* = 50 μm and the cell’s angular position *φ*. The track exhibits periods of directionally persistent as well as periods of localized, erratic motion. The perimeter of the ring is 314 μm. The shown cell performs more than one full revolution. (**b**) Distance covered along the tangential direction of the ring-shaped lane (top), tangential component of cell velocity (middle), and classification into run and rest states (bottom) for the track shown in (**a**). The bimodal analysis reveals the run (blue) and rest (red) periods in the cell track. (**c**) Expanded view highlighting the different character of the trajectory in run (blue) and rest (red) periods. (**d**) Distribution of the mean velocity in the tangential direction, 〈*v*〉_*state*_, within each run and rest period for more than 200 MDA-MB-436 cells. (**e**) Log-linear plot of the survival function, *S*(*t*) = *P*(*T* > *t*), of the persistence times of the run (blue) and rest (red) states. For both states, *S*(*t*) is fitted by an exponential distribution (dashed lines).

Analysis of the morphology of MDA-MB-436 cells moving in the circular lanes predominantly revealed three distinct and characteristic shapes: (i) a polarized, migratory form with one lamellipodium at the anterior end and a tapered posterior end, (ii) a more symmetric morphology with a lamellipodium extending from each end, and (iii), in very rare cases, a shortened appearance exhibiting no lamellipodium at all. By applying a nuclear stain to track the positions of individual cells on the pattern, we found that these phenotypes tend to correlate with distinct migratory behaviors. The polarized morphology was mostly observed during periods in which cells exhibited rapid and directionally persistent motion, whereas phases of symmetric lamellipodium formation or none at all were often typified by non-persistent and erratic cell movements (Supplementary Movie S1). Extended periods showing these distinct phases of directional migration and localized random motions are discernible in a typical cell track, as illustrated in Fig. 2a,c. When a cell’s position along the arc length, *d*_arc_, is plotted over time, the bimodal nature of this behavior becomes evident in the form of run phases which exhibit a continuous slope in *d*_arc_(*t*) and phases of random motion where *d*_arc_(*t*) shows no, or no sustained, slope (Fig. 2b).

In order to quantify this bimodality in cell motion, we performed a statistical analysis that discriminates run from rest states. To this end, we implemented an iterative change-point analysis based on cumulative sum (CUSUM) statistics, in order to determine the transition points between the two states (Supplementary Information). Very similar algorithms have been used to determine trends in the fields of climate research or healthcare^49^,^50^,^51^. Here, we apply the analysis to detect time points at which the fundamental trend of the migration velocity changes. Once these change points are found, the motion between two adjacent change points is classified either as a ballistic run state or a more diffusive rest state by evaluating the slope of the mean squared displacement, MSD(*t*), in the corresponding interval (Fig. 2b, for details see Supplementary Information). This analysis allows to seperately assess the velocity of a cell in the run and rest states. Note that due to the restriction to the quasi 1D environment of the ring pattern, only the velocity in tangential direction along the ring is considered throughout the analysis. Figure 2d shows the corresponding velocity distributions for an ensemble of over 200 MDA-MB-436 cells. The velocity distribution of the run states exhibits two peaks, corresponding to clockwise and counterclockwise cell motion, while the velocity of the rest states is distributed around zero. The absolute run velocity, *v*_run_, is evaluated over all run states, *i*, as *v*_run_ = 〈|*v*_*i*_|〉 = 30.2 ± 0.8 μm/h, where *v*_*i*_ indicates the mean velocity along the lane within the corresponding state. Note that *ν*_run_ is considerably larger than the mean cell velocity evaluated over the entire track lengths, *v*_mean_ = 25.2 ± 0.8 μm/h, since rest periods do not contribute. Because *v*_run_ does hence not depend on the directional persistence of cell motion, it provides a more accurate measure for the actual speed of a fully polarized migrating cell than *v*_mean_. To analyze the persistence of the two states, we evaluated the survival functions, *S*(*t*) = P(*T* > *t*), i.e., the proportion of run or rest periods of length *T* that exceed a given time *t*. We find that *S*(*t*) shows exponential decay, *S*(*t*) ∝ e^−*t*/*τ*^, for both states at timescales longer than about 5 h (Fig. 2e). This exponential behavior suggests that the stochastic process underlying the emergence and collapse of directionally persistent cell migration is Poissonian in nature. (Note that on short timescales the deviations from the exponential in the distributions of both states might be attributable to variations in the position of the nucleus within the cell volume rather than to migratory cell displacement). From the exponential fits, we determine the typical persistence times of the states as *τ*_run_ = 13.6 ± 0.5 h and *τ*_rest_ = 6.5 ± 0.2. Hence, the migration of a cell in this setup is characterized by three parameters: the persistence times of the run and rest states, and the run velocity.

### Locomotory behavior at a PEGylated barrier.

**Figure 3:**
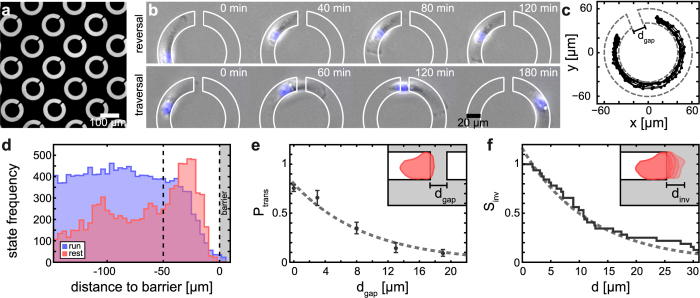
Locomotory behavior at a chemical barrier. (**a**) Fluorescence image of the ring-shaped patterns consisting of labeled fibronectin including a PLL-PEG coated gap of 13 μm width. (**b**) Time series depicting alternative behaviors at the barrier: reversal (top) and traversal of the barrier (bottom). (**c**) Typical trajectory of a cell migrating in ring-shaped lanes interrupted by a 13-μm gap. (**d**) Frequency of cells in the run or rest state plotted against the distance of a 19-μm gap. More than 50 μm away from the barrier most cells are in the run state, while closer than 50 μm to the barrier most cells are in the rest state. (**e**) Probability *P*_trans_ of overcoming a PEG barrier of width *d*_gap_. *P*_trans_ is well fitted by an exponential function (dashed line). (**f**) The ability of cells to penetrate into the PEGylated area at the end of stripes coated with fibronectin is shown by the survival function *S*_inv_(*d*) = *P*(*d*_inv_ > *d*). The dashed line represents an exponential fit.

To explore the influence of chemical barriers on locomotion, we inserted an obstacle in the form of a PEGylated, non-adhesive area of defined width into the circular lane (Fig. 3a). We observed that when a cell encounters the barrier, it either reverses or traverses the barrier (see Fig. 3b and Movies S2, S3). When a cell reverses direction, the polarized morphology characteristic of the run state collapses when it hits the barrier, as the PEGylated area inhibits further progress of the leading edge. The posterior end continues to move forward and the anterior lamellipodium retracts and disappears. As a consequence, polarity is lost and the cell enters the rest state. When the cell repolarizes, the direction away from the barrier is favored since the PEGylated area suppresses formation of protrusions. When considering the distribution of states on the lane, the transition to the rest state at the barrier leads to depletion of run states and accumulation of rest states in the adjacent region, as shown in Fig. 3d. A closer look at the cell projection at the interface shows that the lamellipodium does not stop exactly at the fibronectin/PEG interface, but protrudes into the PEGylated area (Fig. S2). In cases where the lamellipodium succeeds in bridging the whole PEGylated area, the cell can traverse the barrier. We therefore evaluated this transit probability, *P*_trans_(*d*_gap_), i.e. the probability that a cell overcomes the PEGylated barrier, as a function of the barrier width, *d*_gap_. In the ring-shaped geometry, cells can encounter the barrier several times (Fig. 3c and Movie S3) and, together with the parallel acquisition of data from many ring-shaped lanes, this enabled us to study several hundred cell-barrier interactions. The transit probability, *P*_trans_(*d*_gap_), decreases with increasing barrier width and is approximately fitted by an exponential function, *P*_trans_(*d*_gap_) = [1 − *P*_turn_(0)] ⋅ exp(−*d*_gap_/μ_trans_), with a decay length, μ_trans_ = 8.3 ± 2.3 μm, (Fig. 3e). Here, the error indicates the 95% confidence interval within the fit. Note that, in the absence of a barrier, *P*_trans_(0) is less than one due to the fact that there remains a finite probability, *P*_turn_(0) = 1 − *P*_trans_(0), that a cell spontaneously reverses without a barrier present. To explore the origin of the exponential decay of *P*_trans_(*d*_gap_), we use a blind-alley geometry to study the penetration depth of the contour of the leading edge into a PEGylated area with high time resolution (see Fig. S2). We observe cells going into reverse at the fibronectin/PEG interface and derive the maximal invasion depth of the lamellipodium, *d*_inv_. Figure 3f shows the survival function *S*_inv_(*d*) = P(*d*_inv_ > *d*), i.e., the fraction of maximal invasion depths, *d*_inv_, that exceed *d*, which exhibits an exponential decay, *S*_*inv*_(*d*) = *s*_0_ ⋅ exp(−*d*/μ_inv_). The resulting typical invasion depth, *μ*_inv_ = 11.8 ± 1.0 μm, is comparable to the decay length of the transit probability, μ_trans_. Thus, it seems likely that the maximal depth of penetration by the lamellipodium determines the probability that a PEG barrier will be overcome. All observed cells reverse at the end of the blind alley and the highest measured invasion depth of the lamellipodium is about 30 μm. This length might indicate the maximal barrier width that MDA-MB-436 cells are able to overcome. Similar limits have been found for the distance between adhesive points that cells are able to bridge^52^. During the reversal, all cells penetrate at least 2 μm into the PEG area, which is comparable to the distance from the leading edge to the region where the first new focal adhesions are formed^6^,^53^. The experiments show that further penetration of cell protrusions into the PEG area is hampered. This observation indicates that PEGylated surfaces hinder the formation of new focal adhesions. Focal adhesions stimulate actin polymerization in lamellipodia and are therefore needed to form protrusions and maintain cell polarization^4^. Thus, the small number of adhesion points provided on PEG areas^54^ can explain the switch from run to rest state at the fibronectin/PEG interface.

### The migratory fingerprint

**Figure 4:**
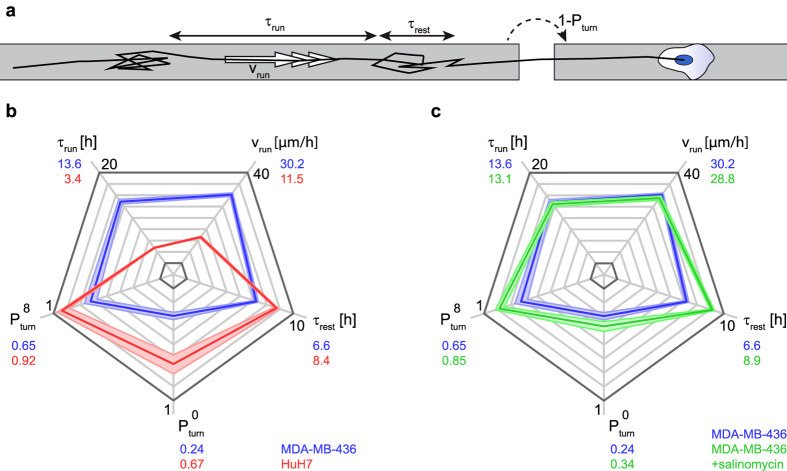
The migratory fingerprint. (**a**) Illustration of the parameters that characterize cell migration in our assay: run persistence time, *τ*_run_, rest persistence time, *τ*_rest_, run velocity, *v*_run_, and reversal probability, 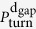, which depends on gap width. (**b**) Migratory fingerprint: When migration parameters are plotted on a radar chart a characteristic polygon is formed. Comparison of MDA-MB-436 and HuH7 cells reveals a lower run velocity, and a lower run persistence, but higher reversal probability and increased resting time for the HUH-7 cell line. The higher overall motility of the MDA-MB-436 cells becomes evident from the upward shift of the polygon. (**c**) Comparison of the migratory behavior of MDA-MB-436 cells in the presence or absence of 50 nM salinomycin. Mean run velocity and persistence of the run state remain almost constant, while the persistence of the rest state is increased. Furthermore, salinomycin increases the reversal probabilities with and without barrier. The decrease in overall motility in the presence of salinomycin is revealed by the downward shift of the polygon.

The bimodal analysis of cell motion on the ring-shaped micropatterns yields three characteristic parameters: *v*_run_, *τ*_run_, and *τ*_rest_ (Fig. 4a). The mean run velocity, *v*_run_, describes the velocity of a polarized migrating cell. Likewise the run persistence time, *τ*_run_, and the rest persistence, *τ*_rest_, are measures for the characteristic lifetimes of the run and rest states, respectively. Additionally, we chose *P*_turn_(8) = 1 − *P*_trans_(8) as a standardized measure to quantify a cell’s ability to invade cell-repelling surface areas. The width of *d*_*gap*_ = 8 μm was chosen such that traversal as well as reversal events are frequently observed. To quantify and compare the reversal probability in the absence of a barrier we, evaluate *P*_turn_(0). Together, this set of parameters provides a characteristic signature of cell migration behavior. This fingerprint-like signature can be illustrated using radar charts similar to the ones used to visualize morphological and structural parameters^55^. In Fig. 4b, the migratory parameter set for the mesenchymal MDA-MB-436 cells is compared to that for HuH7 cells, which are more epithelial in character. The more motile phenotype of MDA-MB-436 relative to HuH7 is evident from the longer run persistence and the considerably higher run velocity, the rest state persistence of the mesenchymal MDA-MB-436 cells is shorter than that for HuH7 cells (see Movie S4). Remarkably, the reversal probability, *P*_turn_(8), at PEGylated surfaces is substantially larger for HuH7 than for MDA-MB-436 cells. This finding is in agreement with the invasive behavior of MDA-MB-436 cells reported in literature in contrast to the less invasive HUH7 cell line^22^,^56^,^57^. In the radar plot, the axes are arranged in such a way that for the more motile MDA-MB-436 cells the polygon is shifted to the top, whereas for HuH7 cells, which are less motile and have a low traversal capability, the polygon is shifted downwards.

The multiparameter characterization also provides a more refined assessment of how drugs affect cell motility. To demonstrate its utility, we study the effect of salinomycin on the five migratory parameters of MDA-MB-436 cells. In previous studies it was found that salinomycin reduces mean velocity and directionality of cells migrating on 2D surfaces^22^. Here, we find that salinomycin-treated cells are less likely to invade PEGylated surfaces and show an increased resting time. However, salinomycin has almost no effect on run persistence or average run velocity (Fig. 4c, see Movie S5). This finding indicates that salinomycin does not interfere with the molecular machinery propelling the cell. Instead, salinomycin reduces the probability that cells will enter the polarized state, as reflected in the extended rest time. Thus, the bimodal description yields a more precise picture of the effect of salinomycin. Indeed, the finding that salinomycin primarily increases the rest time is supported by the observation that exposure to salinomycin leads to an influx of calcium ions into the cytoplasm^58^,^59^, what is likely to affect cell polarization^60^. The multiparameter assessment is hence well suited to derive a fingerprint like characterization of the migratory behavior of different cell types, which can be conveniently characterized graphically by the shapes of the polygons in the radar-plot representation.

## Conclusion

In this work we used ring-shaped micropatterns as a tool for multiparameter characterization of single-cell motion. We found strong evidence for a pronounced bimodality in both phenotypic morphology and migratory kinetics. The two-state analysis employed here yielded a well-defined set of parameters that characterize the migratory properties of individual cells. By adding a PEGylated area to each ring-shaped lane, we were able to investigate the impact of chemical barriers, thus quantifying the sensitivity of a migrating cell to abrupt changes in substrate chemistry. We showed that this set of parameters can clearly discriminate between the motility patterns of different cell lines, as well as quantifying the effects of motility-affecting drugs like salinomycin. In addition, the parameters obtained from standardized migration assays can be used for parameter estimation and validation of mathematical models of cell migration. In future studies, the fingerprint like classification scheme used here can be extended by additional parameters describing cellular behavior and morphology. In this regard, the barrier setup could be modified using customized interfaces composed of different ECM components. By this means, the relationship between the *in vitro* penetration depth into various kinds of ECM-coated areas and the *in vivo* invasiveness of cells could be scrutinized and used for cell testing. In this respect, ring-shaped microlanes with chemical barriers can complement existing migration studies and lead to improved cancer-cell classification and more sophisticated drug-screening assays. Additionally, patterning approaches capable to alter the guidance cues provided by the confinement dynamically, could be applied to analyze and include the cell response to changing external stimuli. Hence, migration assays based on micropatterns, in combination with high-throughput time-lapse acquisition and automated cell tracking, are likely to be of value as standardized platforms for the assessment of single-cell migration and the development of phenotypic descriptors.

## Methods

### Micropatterning

#### Production of stamps

To produce stamps for micro-contact printing as a master for stamp preparation, silicon wafers were coated with TI Prime adhesion promoter and AZ40XT (MicroChemicals) photo-resist. Desired areas were exposed to UV light using laser direct imaging (Protolaser LDI, LPKF). The photoresist was then developed (AZ 826 MIF, MicroChemicals) and silanized (Trichloro(1H,1H,2H,2H-perfluoro-octyl)silane, Sigma-Aldrich). To create the stamp, polydimethylsiloxane (PDMS) monomer and crosslinker (DC 184 elastomer kit, Dow Corning) were mixed in a 10:1 ratio, poured onto the stamp master, degassed in a desiccator, and cured overnight at 50 °C. (Note that masters for stamp preparation can also be created by established protocols, such as those provided by photoresist producers like MicroChem.).

#### Microcontact printing

Microcontact printing was used to produce fibronectin-coated ring-shaped lanes. PDMS stamps were activated with UV light (PSD-UV, novascan) for 5 min. Then, the stamps were incubated for 45 min in a solution containing 40 μg/ml fibronectin (Yo proteins) and 10 μg/ml fibronectin labeled with Alexa Fluor 488 (Life Technologies) dissolved in ultrapure water. Next, stamps were washed with ultrapure water, dried and placed on a petri dish (μ-Dish, Ibidi), which had been activated with UV light for 15 min. A droplet of a 1 mg/ml poly-L-lysine-grafted polyethylene glycol (PLL-PEG) (2 kDa PEG chains, SuSoS) solution (dissolved in 10 mM HEPES containing 150 mM NaCl was placed at the edge of the stamps and drawn into the spaces between surface and stamp by capillary action. Stamps were removed and a glass coverslip was placed on the dish surface to ensure complete coverage of the surface with PEG solution. After a 30-min incubation, the coverslip was removed and the surface was washed three times with phosphate-buffered saline (PBS) and stored in PBS until cells were seeded. An area of up to 1.5 cm^2^ was patterned, resulting in up to 5000 ring patterns per dish.

### Cell Culture

MDA-MB-436 breast cancer cells were cultured in DMEM-F12 medium (c.c.pro) containing 10% fetal calf serum (FCS) (Invitrogen) and 2.5 mM L-glutamine (c.c.pro). HuH7 cells were cultured in RPMI medium (c.c.pro) containing 10% FCS (Invitrogen), 2 mM L-glutamine, 5 mM HEPES, and 1 mM sodium pyruvate (c.c.pro). Cells were incubated at 37 °C in a 5% CO_2_ atmosphere. For time-lapse experiments, about 10,000 cells were seeded per dish (μ-Dish, ibidi). After 2 h, cell medium was replaced by 1 ml L15 medium (c.c.pro) containing 25 nM Hoechst 33342 dye (Invitrogen).

### Time-Lapse Microscopy

Scanning time-lapse measurements were performed using an automated inverted microscope (iMIC, Till Photonics) with a 10x Zeiss objective, a Oligochrome lamp (Till Photonics) and an ORCA-03G camera (HAMAMATSU). Cells were maintained at 37 °C using a temperature-controlled mounting frame (ibidi temp. controller, ibidi). Phase-contrast and fluorescent images were automatically acquired every 10 min. To analyze membrane protrusions at the interface of the micropatterns, a 40x objective was used and bright-field images were acquired every 5 sec.

### Cell Tracking

Cell tracking was performed using the image-processing software ImageJ. Isolated cells confined in the ring-shaped lanes were identified by eye. Fluorescence images of the nuclei were preprocessed by applying a bandpass filter and a threshold for fluorescence intensity, and the centers of mass of the stained nuclei were identified. Cell tracks were truncated in the case of cell division or when cells migrated out of the pattern. Cell tracks shorter than 18 h, as well as tracks of dead or non-moving cells were excluded from further analysis.

### Data Analysis

Track analysis was performed in MATLAB (Mathworks). A circle was fitted to cell position data to find the center of the ring-shaped lane. Switching to polar coordinates, the tangential component of the cell velocity was evaluated as *ν*_*i*_ = *R* · (φ_*i*+1_ − *φ*_*i*_), where *φ*_*i*_ indicates the azimuthal cell position at time *i* and *R* indicates the mean radius of the micropattern (*R* = 50 μm). To distinguish run from rest states, a change-point analysis in combination with a characterization of cell dynamics via evaluation of the mean square displacement was applied. (see Supplementary Information)

### Evaluation of Parameters Characterizing Cell Migration

To identify characteristic parameters of cell motion, 211 MDA-MB-436 cells, 162 MDA-MB-436 cells treated with 50 nM salinomycin, and 101 HuH7 cells were tracked and analyzed. We evaluated the following measures:

***ν***_**run**_: The run velocity of a cell population is defined as the mean over the velocities of each individual run state *v*_*run*_ = 〈|〈*v*〉_*state i*_|〉_*i*_. The error range given is the standard error of the mean.

***τ***_**run**_, ***τ***_**rest**_: To evaluate the persistence times of run and rest states *τ*_run_ and *τ*_rest_, the survival function *S*(*t*) = P(*T* > *t*) is calculated. *τ*_run_ and *τ*_rest_ are determined by fitting log(*S*(*t*)) by the function 
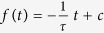
 in the range where *S*(*t*) shows clearly exponential behavior (i.e >2.5 h (rest state) or >5 h (run state)). To assure that the resulting values are not biased by the limited time for which the cells are observed, only states that start at least 20 h before the end of the corresponding cell track are evaluated, while the fitting range for *S*(*t*) ends at 20 h. The error range given is the 99% confidence interval for the fit.

***P***_**turn**_(***d***_**gap**_): Turning probabilities for the given barrier width, *d*_gap_, are determined by evaluating how often cells go into reverse after the nucleus enters the region within 50 μm of the barrier. So *P*_turn_(*d*_gap_) corresponds to the number of times a cell leaves the region on the same side as it entered, divided by the number of times the region was entered. *P*_turn_(0) is the mean value of spontaneous turning probabilities at 8 arbitrary 50-μm regions in a ring-shaped lane without any barrier. The error range given is the Clopper-Pearson 95% confidence interval for binomial distributions. For barrier widths of 3, 8, 13 and 19 μm, 158, 265, 209 and 296 barrier encounters of MDA-MB-436 cells were evaluated, respectively. A further 303 encounters of salinomycin-treated MDA-MB-436 cells and 101 encounters of HuH7 cells with an 8-μm barrier were analyzed.

## Additional Information

**How to cite this article**: Schreiber, C. *et al.* Ring-Shaped Microlanes and Chemical Barriers as a Platform for Probing Single-Cell Migration. *Sci. Rep.* **6**, 26858; doi: 10.1038/srep26858 (2016).

## Supplementary Material

Supplementary Movie S1

Supplementary Movie S2

Supplementary Movie S3

Supplementary Movie S4

Supplementary Movie S5

Supplementary Information
