# Supplementary material for: Ring-Shaped Microlanes and Chemical Barriers as a Platform for Probing Single-Cell Migration
Source: Sci Rep. 2016 May 31;6:26858. doi: 10.1038/srep26858 (PMC4886529; doi:10.1038/srep26858)
Supplement: Supplementary Information [file srep26858-s6.pdf]

Ring-Shaped Microlanes and Chemical Barriers as a Platform for Probing Single-Cell Migration

Supplementary Material

Christoph Schreiber[1†], Felix J. Segerer[1†], Ernst Wagner[2],
Andreas Roidl[2] and Joachim O. Rädler[1*]

Faculty of Physics and Center for NanoScience, Ludwig-Maximilians-Universität München, Geschwister-Scholl-Platz 1, D-80539 Munich, Germany

[2] *Department of Pharmacy, Center for System-based Drug Research, Ludwig-Maximilians Universität München, Butenandtstraße 5-13, Building D, 81377 Munich, Germany*

[†] C.S. and F.J.S. *contributed equally to this work*

[*] *To whom correspondence should be addressed: J.O. Rädler, E-mail: raedler@lmu.de*
Phone: +49-(0)89-2180-2438 Fax: +49-(0)89-2180-3182

S1.Bimodal Classification of Cell Motion

In order to evaluate the transition points between the run and rest states, we implemented an iterative change point (CP) analysis based on classical cumulative sum (CUSUM) statistics in combination with a motion classification via a fit of the mean squared displacement (MSD) (Fig. S1).

To this end, we first calculate the CUSUM of the angular velocity $\omega(t)$ for each time *t* within a track:

$$S(t) = \sum_{i=1}^{t} [\omega(t) - \bar{\omega}] \tag{1}$$

Here, $\bar{\omega}$ denotes the average velocity within the tracking interval $t = 0,\ldots,T$. Note that the velocity, v, introduced in the main text is calculated via $v = R \cdot \omega$ where the constant *R* indicates the average lane radius. To evaluate whether a CP occurred within the analyzed interval, we estimate a confidence level for the CP existence via a bootstrap analysis. To this end, we define the estimator for the magnitude of the change as

$$S_{\text{diff}} = [\max_{t=0,\ldots,T} S(t)] - [\min_{t=0,\ldots,T} S(t)], \tag{2}$$

which is calculated for the CUSUM of the actually measured velocity as well as for a set of bootstrapped CUSUMs of which the consecutive order of $\omega(t) : t \in \{0,\ldots,T\}$ is permutated

randomly (Fig.S1b). The confidence level for at least one CP occurrence within the interval is now calculated as

$$L_{\mathrm{conf}} = \frac{N_{(S_{\mathrm{diff}} < S^0_{\mathrm{diff}})}}{N_{\mathrm{perm}}}. \tag{3}$$

Here, $N_{(S_{\mathrm{diff}} < S^0_{\mathrm{diff}})}$ denotes the number of bootstrap CUSUMs for which S_{diff} is smaller than the estimator of the original CUSUM, S^0_{diff}, and N_{perm}denotes the total number of bootstrap samples. If not denoted otherwise we chose $N_{perm} = 10000$. If L_{conf} is above a certain threshold level L_{th} we evaluate the position of the CP via the CUSUM estimator:

$$S_{\mathrm{CP}} = \max_{t=0,\ldots,T} |S(t)| \tag{4}$$

As a trade-off between accuracy and liability to small fluctuation in cell motion we choose $L_{\mathrm{th}} = 0.7$. If a CP is detected with confidence $L_{\mathrm{conf}} > L_{\mathrm{th}}$, this procedure is repeated iteratively for the two resulting intervals adjacent to the CP until no further CPs with a confidence level $L_{\mathrm{conf}} > L_{\mathrm{th}}$ are detected.

In the next step, to determine if an interval is part of the running or resting period of a cell, the time averaged MSD is calculated for each interval between two consecutive CPs via:

$$\mathrm{MSD}(t) = \frac{1}{T-t+1} \sum_{\tau=0}^{T-t} [\varphi(\tau+t) - \varphi(\tau)]^2 \tag{5}$$

The MSD is now fitted by a fitting function $f(t) = a \cdot t^b$ where the fitting exponent b indicates whether a motion pattern shows normal ($b = 1$) or anomalous ($b \neq 1$) diffusion. Here, a value of $b = 2$ would correspond to ballistic motion. In order to identify periods where a cells show directional persistent motion, we choose a threshold of $b_{\mathrm{th}} = 1.75$ classifying all intervals where $b < b_{\mathrm{th}}$ as non ballistic and therefore rest states and all intervals where $b \geq b_{\mathrm{th}}$ as ballistic and consequently run state periods. Note that for very short intervals (T < 60 min) where only a very limited set of data points is accessible, the fit of the MSD is not a very robust measure. Hence for such intervals we choose the criterion such that if $\omega(t)$ is strictly increasing or decreasing within the interval, the interval is considered a run state, while it is considered a rest state if this condition is not met. Finally, all CPs between two rest state periods or two run state periods in which the cell moves in the same direction are removed.

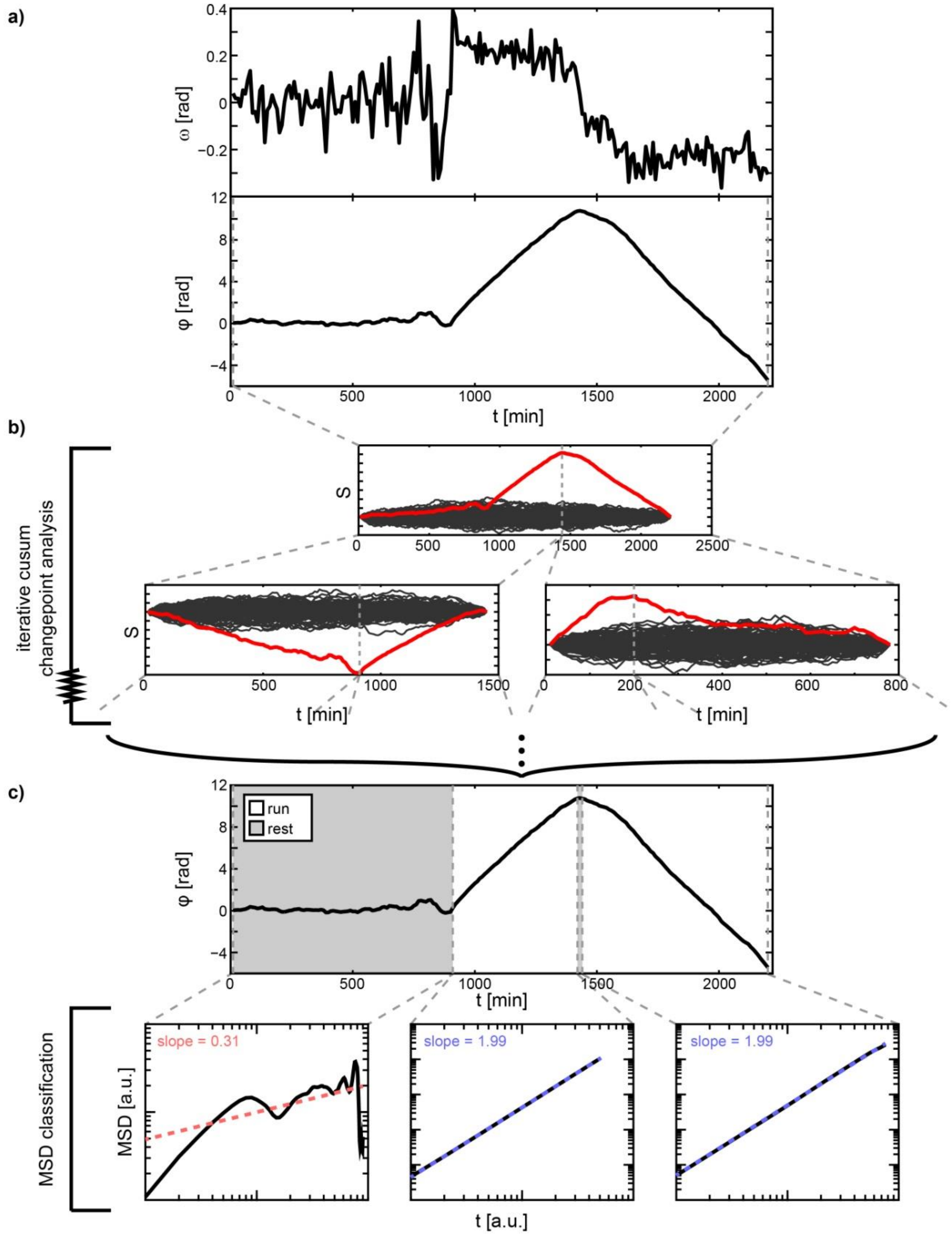

Figure S1. Illustration of the bimodal data analysis. (**a**) Time evolution of the azimuthal position, $\boldsymbol{\varphi(t)}$, (with respect to the initial position $\boldsymbol{\varphi(0) = 0}$) and the angular velocity, $\boldsymbol{\omega(t)}$, of an exemplary cell migrating on a ring shaped micropattern. (**b**) Via an iterative CP analysis based on CUSUM statistics, characteristic changes in the trend of $\boldsymbol{\omega(t)}$ are identified. Therefore, to estimate the existence and eventual position of a CP, the CUSUM, $\boldsymbol{S(t)}$,of $\boldsymbol{\omega(t)}$ (red) is evaluated and compared to bootstrapped CUSUMs of the same time period (black). This procedure is repeated iteratively for the resulting intervals prior to and after a detected CP until no further CPs are detected. (**c**) The character of the cell motion within the found intervals is identified by evaluating and fitting the corresponding MSD. The MSDs (black) are plotted in log-log representation and fitted by a function $\boldsymbol{f(t) = a \cdot t^b}$ (red/blue). In the log-log representation the slope $\boldsymbol{b}$ of the fit function indicates the type of cell motion (rest/run).

S2. Cell Invasion of PEGylated Areas

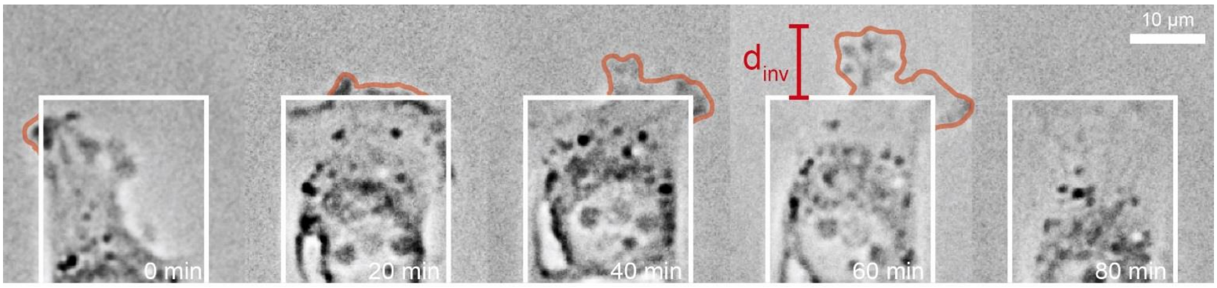

Figure S2. Time lapse series of a MDA-MB cell turning around at the end of a blind alley geometry. The cell's protrusions invade the surrounding PEG area (indicated in red). The maximal invasion depth d_{max} is determined manually for 32 encounters of cells with the fibronectin PEG interface and the resulting survival function $S(d_{\text{inf}})$ is shown in Fig. 3f.

Description of Supplementary Movies

Movie S1: MDA-MB 436 cell migrating on a ring shaped micropattern. The cell exhibits a bimodal motion pattern alternating between run and rest state. The track of the cell is shown in Fig. 2.

Movie S2: MDA-MB 436 cell migrating on a ring shaped micropattern with a PEGylated barrier of 13 µm in width. At the barrier the cell shows reversal as well as transversals.

Movie S3: MDA-MB 436 cell migrating on a ring shaped micropattern with a PEGylated barrier of 13 µm in width. The cell repeatedly reverses direction at the barrier.

Movie S4: HuH7 cell migrating on a ring shaped micropattern.

Movie S5: MDA-MB 436 treated with 50 nM salinomycin migrating on a ring shaped micropattern.
